# Role of F-18 FDG PET/CT in non-conjunctival origin ocular adnexal mucosa-associated lymphoid tissue (MALT) lymphomas

**DOI:** 10.1186/s13550-019-0562-1

**Published:** 2019-11-21

**Authors:** Hye Lim Park, Joo Hyun O, Sonya Youngju Park, Seung-Eun Jung, Gyeongsin Park, Byung-Ock Choi, Sung Hoon Kim, Young-Woo Jeon, Seok-Goo Cho, Suk-Woo Yang, On behalf of Catholic University Lymphoma Group

**Affiliations:** 1 Division of Nuclear Medicine, Department of Radiology, Eunpyeong St. Mary’s Hospital, College of Medicine, The Catholic University of Korea, Seoul, Republic of Korea; 2 Division of Nuclear Medicine, Department of Radiology, Seoul St. Mary’s Hospital, College of Medicine, The Catholic University of Korea, 222, Banpo-daero, Seocho-gu, Seoul, 06591 Republic of Korea; 3 Division of Nuclear Medicine, Department of Radiology, Yeouido St. Mary’s Hospital, College of Medicine, The Catholic University of Korea, Seoul, Republic of Korea; 4 Department of Radiology, Eunpyeong St. Mary’s Hospital, College of Medicine, The Catholic University of Korea, Seoul, Republic of Korea; 5 Department of Hospital Pathology, Seoul St. Mary’s Hospital, College of Medicine, The Catholic University of Korea, Seoul, Republic of Korea; 6 Department of Radiation Oncology, Seoul St. Mary’s Hospital, College of Medicine, The Catholic University of Korea, Seoul, Republic of Korea; 7 Department of Hematology, Yeouido St. Mary’s Hospital, College of Medicine, The Catholic University of Korea, Seoul, Republic of Korea; 8 Department of Hematology, Seoul St. Mary’s Hospital, College of Medicine, The Catholic University of Korea, Seoul, Republic of Korea; 9 Department of Ophthalmology, Seoul St. Mary’s Hospital, College of Medicine, The Catholic University of Korea, 222, Banpo-daero, Seocho-gu, Seoul, 06591 Republic of Korea

**Keywords:** Non-conjunctival MALT lymphoma, FDG PET/CT, staging

## Abstract

**Background:**

Despite the widespread use of F-18 fluorodeoxyglucose positron emission tomography/computed tomography (FDG PET/CT) in the diagnosis and response assessment of patients with lymphoma, few studies have assessed its value in ocular adnexal lymphomas. The purpose of this study was to evaluate the role of FDG PET/CT in staging of non-conjunctival origin ocular adnexal mucosa-associated lymphoid tissue (MALT) lymphomas (OAML). In addition, the diagnostic sensitivity of FDG PET/CT was compared with magnetic resonance imaging (MRI). FDG PET/CT of 123 consecutive patients with pathologically proven OAML between January 2009 and February 2016 were retrospectively reviewed. The patients with MALT lymphoma originating from conjunctiva were excluded. A total 50 patients with non-conjunctival origin OAML were assessed. Maximum standardized uptake value (SUVmax) and additional PET parameters were measured for all lesions. Sensitivity for primary tumor detection was compared with MRI.

**Results:**

Ten patients had bilateral OAML and total 60 OAML lesions were analyzed. MRI was missing in one patient. The tumor locations were as follows: eyelid, 9; lacrimal gland, 18; orbit, 33. Fifty lesions (83.3%) were FDG-avid tumors with mean ± SD SUVmax 4.8 ± 2.4 (range 2.0~11.1). The mean SUVmax according to tumor location were as follows: eyelid, 3.7 ± 1.1 (2.8~5.3); lacrimal gland, 3.6 ± 1.4 (2.1~6.4); orbit, 5.7 ± 2.6 (2.0~11.1). Mean SUVmax among the 3 locations were statistically different (*P* = 0.010). The sensitivity was calculated as 83.1% (49/59) for FDG PET/CT and 89.8% (53/59) for MRI, which were statistically comparable (*P* = 0.219 by McNemar’s test). Seven of 50 patients (14%) were upstaged by detection of extraocular lesions by FDG PET/CT (1 kidney and lung, 1 tonsil, 4 cervical LNs, 1 sacral foramen).

**Conclusion:**

83.3% of the non-conjunctival origin OAML were FDG-avid tumors, with FDG PET/CT showing comparable sensitivity to that of MRI. FDG PET/CT detected unsuspected extraocular lymphoma involvement in 14% of the patients. FDG PET/CT performed for staging of non-conjunctival origin OAML may thus guide therapeutic management.

## Background

Ocular adnexal lymphoma (OAL) is the most common ocular tumor and constitutes 1% of non-Hodgkin lymphomas (NHL) and 8% of extranodal lymphomas [1]. The most common subtype of OAL is mucosa-associated lymphoid tissue (MALT) lymphomas, and the incidence of ocular adnexal MALT lymphoma (OAML) is high in Japan and Korea (80~98%) compared with Western countries (50~78%) [1–4]. Primary OAML involves conjunctiva, lacrimal gland, eyelid, and orbit. Most of primary OAML patients show localized disease (stage IE) at diagnosis and nodal involvement (5%) or systemic involvements (10–15%) are uncommon [1]. However, nodal involvement or systemic involvements are poor prognostic factors, and treatment beyond local radiation is needed for systemic disseminated disease [5].

F-18 fluorodeoxyglucose positron emission tomography/computed tomography (FDG PET/CT) is widely used in the diagnosis and response assessment of patients with Hodgkin lymphomas and NHL [6–8]. However, few studies have assessed its value in OAL, since MALT lymphoma is low-grade B cell NHL and often shows low FDG-avidity on FDG PET/CT [7, 8]. A wide range of detection rates of FDG PET/CT in MALT lymphoma is reported related to the variability of FDG-avidity of MALT lymphoma [9]. Tumor stage, location, morphologic features, and Ki-67 index were factors that determined the FDG-avidity in MALT lymphoma [10, 11].

Orbit is the most common site of OAML (40%), and conjunctiva is another often involved site (35~40%). Incidence of lacrimal gland and eyelid tumors are 10~15% and 10%, respectively [1]. The incidence of conjunctival OAML is as high as orbit. However, most of the patients underwent PET/CT after excision or biopsy of conjunctival mass. Also, physiologic activity of conjunctiva, small tumor volume, and relatively low FDG-avidity all make it difficult to interpret FDG uptake of conjunctival lesions. Clinically non-conjunctival lymphoma is reported to have higher rate of extraocular involvement and worse prognosis compared with conjunctival lymphoma [5, 12], and thus may require more aggressive treatment strategy.

The purpose of this study was to evaluate the role of FDG PET/CT in staging of non-conjunctival origin OAML, and to compare its diagnostic sensitivity to magnetic resonance imaging (MRI).

## Materials and methods

### Patient population

**Fig. 1:**
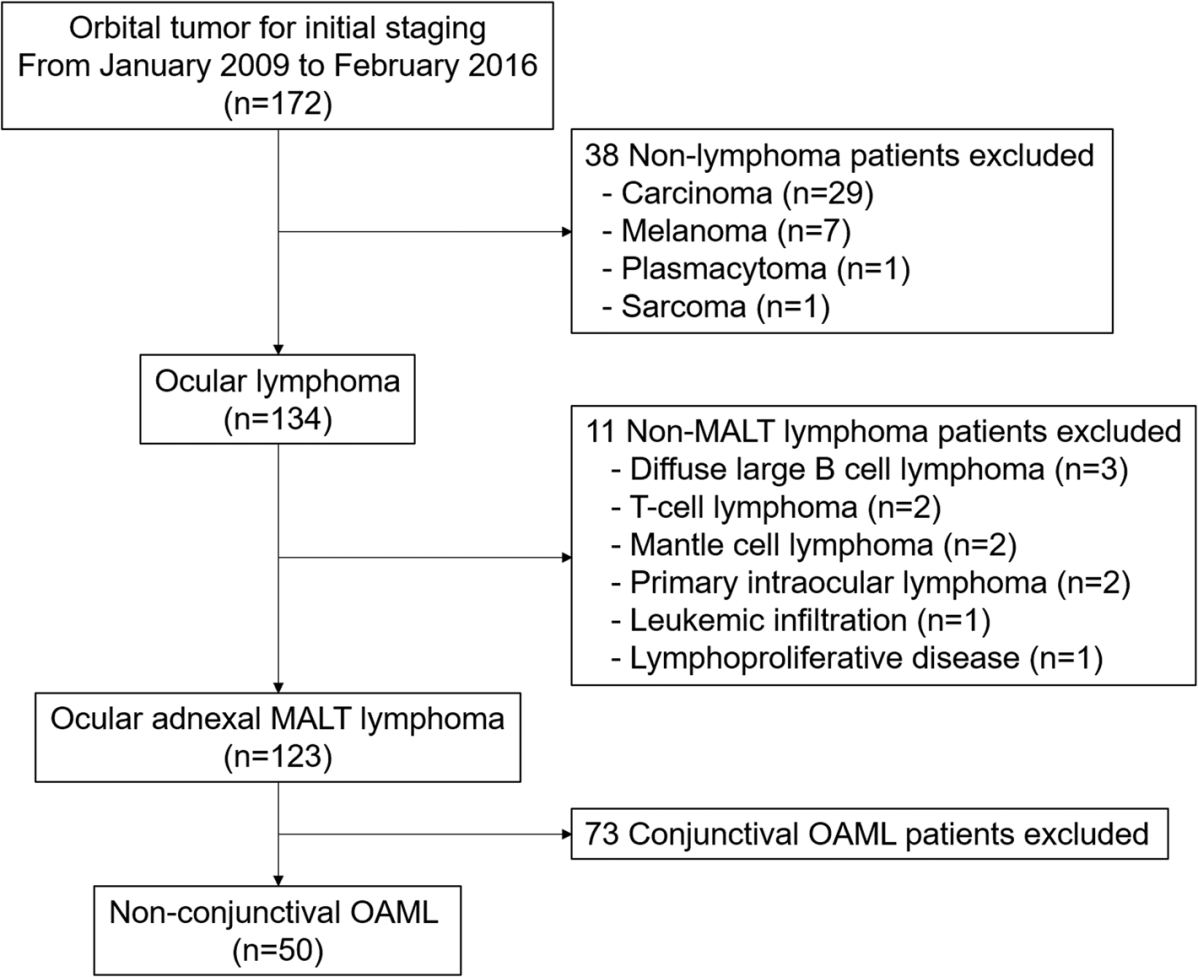
Flowchart of study population

We reviewed clinical records of patients with pathologically confirmed MALT lymphomas. A total of 123 consecutive patients with OAML had pre-treatment FDG PET/CT scans between January 2009 and February 2016. Seventy-three patients with MALT lymphoma involving the conjunctivae alone were excluded. A total of 50 patients with non-conjunctival origin OAML were assessed (Fig. 1). This retrospective study was approved by Catholic Medical Center Institutional Review Board. Informed consent was waived.

### FDG PET/CT protocol

All patients fasted for at least 6 h before the FDG PET/CT scan. An amount of 3.7~5.5 MBq/kg FDG was injected intravenously, and scanning began 60 min later. No intravenous contrast agent was administered. Images were acquired using a combined PET/CT in-line system (Biograph Duo, Biograph Truepoint, Biograph mCT, Siemens Medical Solutions; Discovery 710D, GE Healthcare). The acquisition time was 2~3 min per bed position. All patients were in the supine position during PET/CT scanning. Non-contrast-enhanced low-dose CT began at the orbitomeatal line and progressed to the proximal thigh using a standard protocol: 130 kVp, 80 mAs, 5-mm slice thickness (Biograph Duo); 120 kVp, 50 mAs, 5-mm slice thickness (Biograph TruePoint); 100~120 kVp, variable mAs adjusted by topographic image, 3-mm slice thickness (Biograph mCT); and 120 kVp, variable mAs adjusted by topographic image, 2.5-mm slice thickness (Discovery 710D). PET scans of the same body region followed immediately. The CT data were used for attenuation correction, and images were reconstructed using a standard ordered-subset expectation maximization algorithm.

### Orbital MRI protocol

MRI scans were acquired with the patient in a prone position in a 3.0-T scanner (Magnetom Verio, Siemens Medical Solutions) equipped with an orbit coil using the following sequences: axial, turbo spin-echo T2-weighted imaging sequence and fat suppressed, pre- and post-contrast axial T1- weighted sequences obtained before and after Gd-DTPA injection.

### Image analysis

FDG PET/CT scans were centrally reviewed by two nuclear medicine physicians, both with over 10 years of experience in ocular lymphoma PET/CT image interpretation, with knowledge of clinical diagnosis to reach a consensus. FDG PET/CT images were assessed using software, XD3 (Mirada Medical). FDG-avid tumor was defined as tumor showing discrete FDG uptake higher than background soft tissue on visual assessment, and we measured the maximum and mean standardized uptake value (SUVmax, SUVmean), and metabolic tumor volume (MTV). Threshold SUV of 2.0 was used for MTV computation. Total lesion glycolysis (TLG) was also obtained. Orbital MRI data were reviewed for this study by one radiologist aware of the tumor location. On MRI, OAML was defined as soft tissue mass with high signal intensity (SI) on T2-weighted sequence, low SI on T1-weighted sequence, and homogeneous enhancement on gadolinium-enhanced sequence [13].

For the diagnosis of lymphoma involvement at an extraocular lesion detected by PET/CT, histologic confirmation was performed when possible (*n* = 3). In the cases without biopsy confirmation, the final diagnosis was made by imaging and clinical follow-up (*n* = 4). Clinically, lesions showing unequivocal response after systemic chemotherapy were considered positive for lymphoma.

### Statistical analysis

Statistical analysis was carried out using the Statistical Package for Social Sciences (SPSS statistics 24) software. Mann-Whitney *U* test with Bonferroni correction was performed for comparison among SUVmax, SUVmean, MTV, and TLG of primary tumors categorized by location. Sensitivity of PET/CT and MRI were compared by the McNemar’s test. *P* values < 0.05 were considered to indicate statistical significance.

## Results

Fifty patients with non-conjunctival OAML were included in this study (30 male and 20 female, median age 53.5, range 25~80). Ten patients had bilateral OAML (eyelid, 2; lacrimal gland, 5; and orbit, 3), and a total of 60 ocular lesions were assessed. The locations of OAML were as follows: eyelid, 9; lacrimal gland, 18; orbit, 33. Patient characteristics are presented in Table 1.

**Table 1:** Patient characteristics

Characteristics	Number (%)
Median age (range)	53.5 years (25~80)
Sex	
Male	30 (60.0)
Female	20 (40.0)
Ann Arbor stage	
IE-IIE	41 (82.0)
IIIE-IVE	9 (18.0)
LDH^a^	
Normal	46 (92.0)
Elevated	4 (8.0)
ECOG^b^ Performance status	
0–1	50 (100.0)
2–4	0 (0.0)
International prognostic index	
0–1	40 (80.0)
2	9 (18.0)
3	1 (2.0)
4	0 (0.0)
Location of OAML^c^	
Eyelid	7 (14.0)
Lacrimal gland	13 (26.0)
Orbit	30 (60.0)
Laterality	
Unilateral (right: left)	40 (25:15)
Bilateral	10
Initial treatment	
Chemotherapy	37 (74.0)
Radiation	6 (12.0)
Chemotherapy and radiation	6 (12.0)
Excision	1 (2.0)

^a^*LDH* lactate dehydrogenase

^b^*ECOG* Eastern Cooperative Oncology Group

^c^*OAML* ocular adnexal mucosa-associated lymphoid tissue lymphoma

**Fig. 2:**
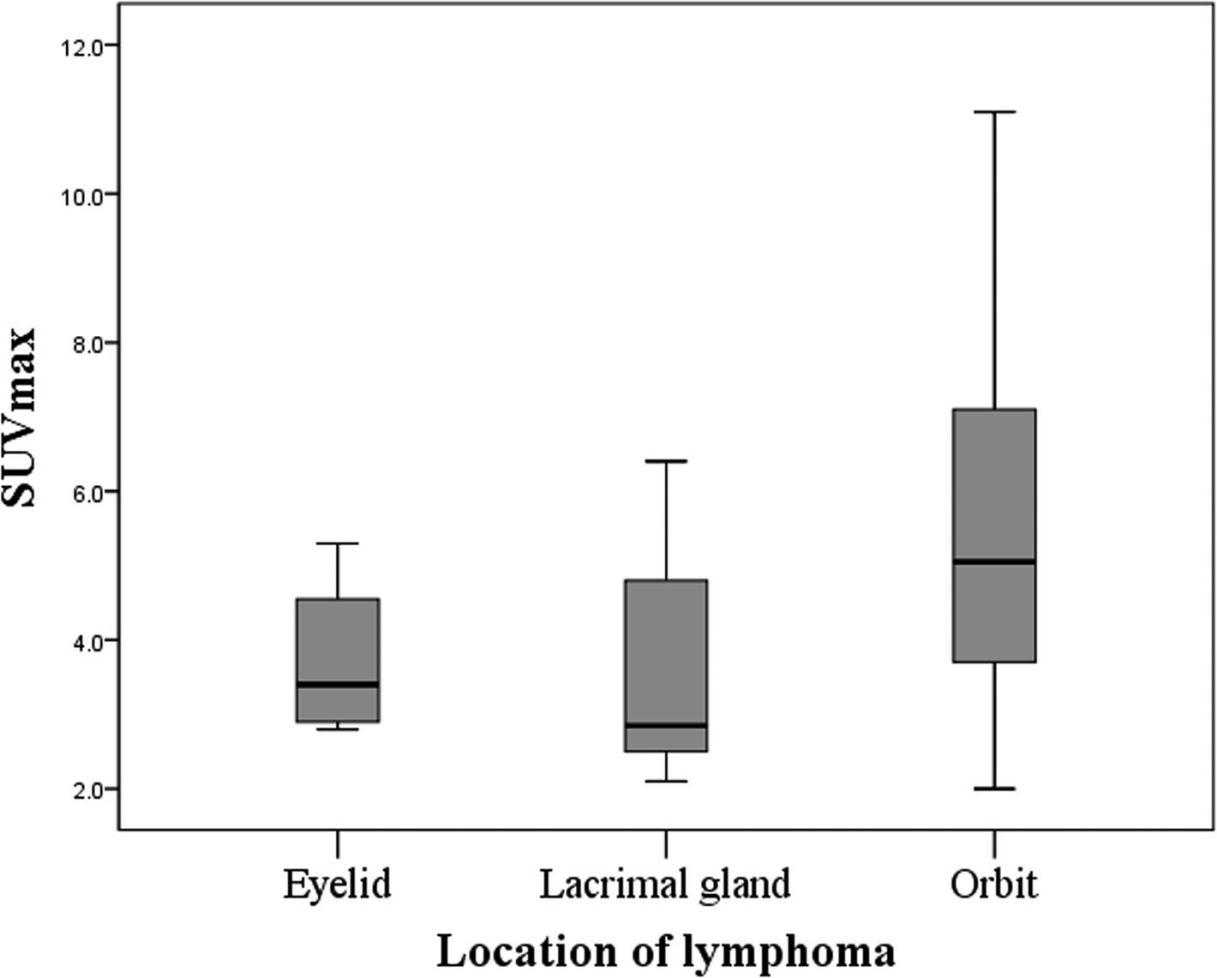
SUVmax according to tumor location

On FDG PET/CT scan, 50 lesions (83.3%) were FDG-avid with mean ± standard deviation (SD) SUVmax of 4.8 ± 2.4 (range 2.0~11.1). Locations of FDG-avid tumors were 4 of eyelid, 18 of lacrimal gland, and 28 of orbit. The mean SUVmax, SUVmean, MTV (cm^3^), and TLG according to tumor location are presented in Table 2. Mean SUVmax (*P* = 0.010), SUVmean (*P* = 0.001), MTV (*P* = 0.015), and TLG (*P* = 0.006) among the 3 locations were statistically different by Kruskal-Wallis test. The differences of each group were calculated by Mann-Whitney *U* test using Bonferroni correction, and mean SUVmax between lacrimal gland and orbit was statistically different (*P* = 0.012) (Fig. 2). Also, mean SUVmean, MTV, and TLG between lacrimal gland and orbit were significantly different (*P* < 0.001, *P* = 0.015, and *P* = 0.006, respectively). There were 10 OAML lesions in nine patients with imperceptible FDG uptake (5 lesions in eyelid and 5 lesions in orbit).

**Table 2:** The mean ± standard deviation and range of PET parameters according to the location of lymphoma

Location	Eyelid	Lacrimal gland	Orbit
SUVmax	3.7 ± 1.1 (2.8~5.3)	3.6 ± 1.4 (2.1~6.4)	5.7 ± 2.6 (2.0~11.1)
SUVmean	2.5 ± 0.7 (1.9~3.5)	2.2 ± 0.7 (1.5~3.8)	3.4 ± 1.2 (1.7~6.4)
MTV (cm^3^)	2.8 ± 1.0 (2.1~4.2)	2.6 ± 2.2 (0.2~7.0)	7.1 ± 6.8 (0.2~26.0)
TLG	7.3 ± 3.5 (4.0~10.5)	7.0 ± 7.4 (0.3~25.1)	28.1 ± 31.7 (0.4~121.7)

MRI was missing in one patient. For the evaluation of sensitivity, 59 lesions were analyzed. On orbital MRI, 53 lesions were detected and the locations were as follows: eyelid, 6; lacrimal gland, 18; orbit, 29. The sensitivity for the ocular lesions was calculated as 83.1% (49/59) for FDG PET/CT and 89.8% (53/59) for MRI, which were statistically comparable (*P* = 0.219 by McNemar’s test).

**Fig. 3:**
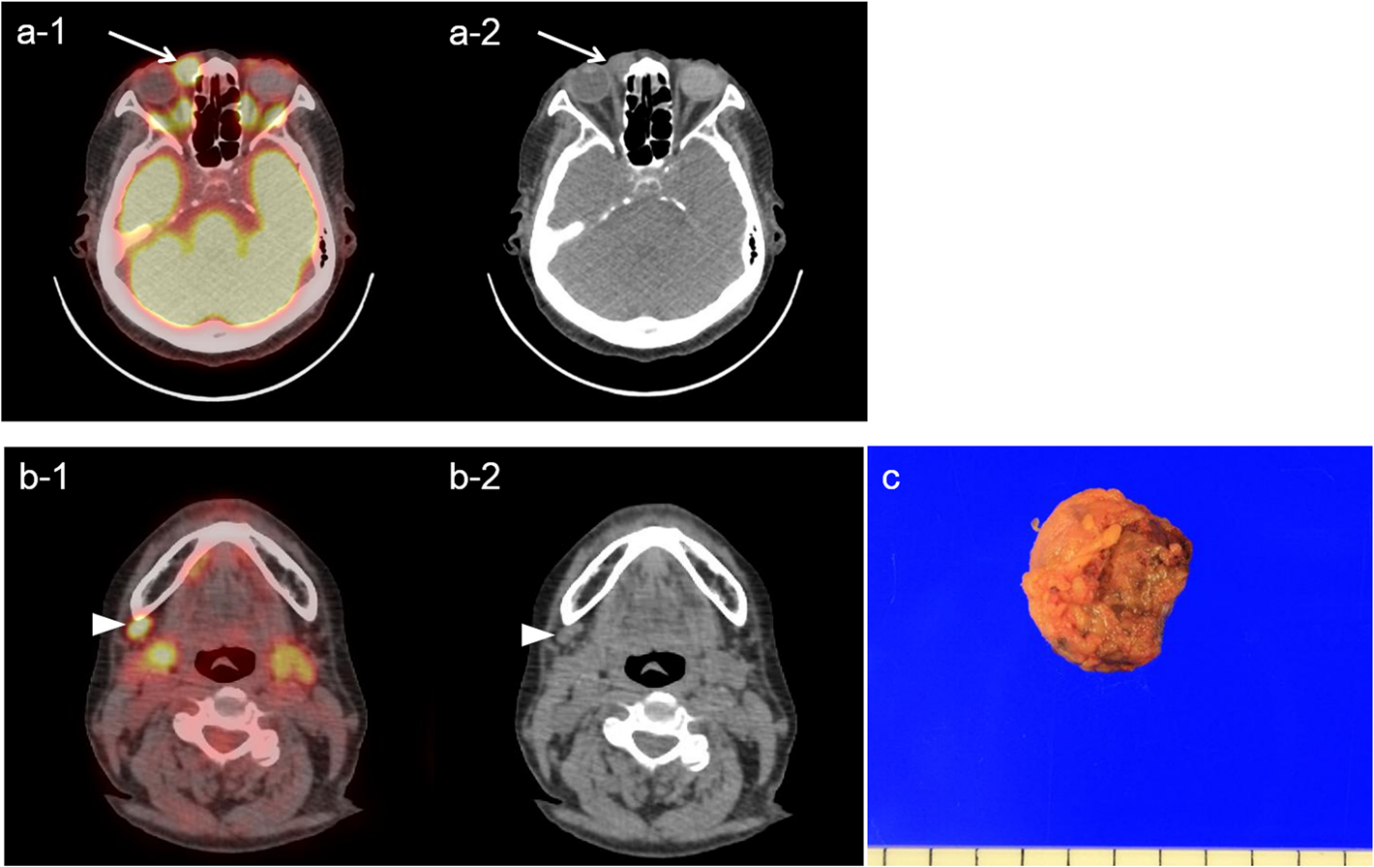
OAML in right orbit (lacrimal sac) with cervical LN involvement. A 72-year-old female had MALT lymphoma in right orbit. **a** Focal FDG uptake was seen in right lacrimal sac (arrows, SUVmax 10.5) on FDG PET/CT scan. **b** Another focal FDG uptake was noted in right cervical level I (arrowheads, SUVmax 10.8). **c** Surgical excision of right cervical level I LN was done, and the final pathology was MALT lymphoma

Seven of 50 patients (14%) were upstaged by detection of extraocular lesions by FDG PET/CT to either IIE or IVE (Fig. 3). Of the seven patients, one patient had positive bone marrow biopsy result, but the 6 other patients were upstaged by FDG PET/CT findings alone. Characteristics of extraocular lesions detected by FDG PET/CT are presented in Table 3. Neck nodes were the most commonly involved extraocular sites.

**Table 3:** Characteristics of extraocular lesions detected in seven patients

Patient No.	Location of OAML^a^	Stage		Locations of extraocular MALT^b^ lymphoma	SUVmax of OAML	SUVmax of extraocular lesions
		Before PET	After PET			
1	Lacrimal gland	IE	IVE	Left kidney^c^	2.7	7.4
				Lung		1.5
				Retroperitoneal lymph nodes		5.5
15	Lacrimal gland	IVE	IVE	Bilateral palatine tonsils^c^	R 2.4; L 3.0	8.0
				Bone marrow		NA^d^
22	Lacrimal gland	IE	IIE	Bilateral cervical nodes	R 4.2; L 2.7	4.9
35	Lacrimal gland	IE	IIE	Right parotid gland	2.1	3.7
				Left submandibular gland		3.6
40	Orbit	IE	IIE	Right cervical level I node^c^	10.5	10.8
47	Orbit	IE	IVE	Sacral foramen	11.0	6.7
				Right parotid gland		3.6
49	Orbit	IE	IIE	Bilateral cervical nodes	R 4.9; L 6.5	5.5

^a^*OAML* ocular adnexal mucosa-associated lymphoid tissue lymphoma

^b^*MALT* mucosa-associated lymphoid tissue;

^c^Pathologically confirmed lymphoma involvement. Other lesions were confirmed by imaging studies and at least 6 months of follow-up

^d^*NA* not available

In this study population, there were 8 patients finally diagnosed with stage IVE: 5 patients had bone marrow involvement confirmed by bone marrow biopsy, and 3 patients had disseminated disease. Of the 3 patients with disseminated disease, one patient had multiple skin lesions without FDG uptake that was confirmed by biopsy. Two patients had highly FDG-avid multi-organ involvement.

## Discussion

Marginal zone lymphoma often clinically presents as extranodal lesions, and rarely presents as nodal disease. OAML is one manifestation of extranodal lymphoma [2, 14]. There were a few studies about the role of FDG PET/CT in diagnosis and staging of OAL [15–18], and the role of FDG PET/CT in treatment response evaluation [17, 19]. To our knowledge, this is the first FDG PET/CT study evaluating the findings and the role of FDG PET/CT in staging of non-conjunctival OAML.

Our institution is a tertiary referral hospital with External Eye Disease Clinic at the Ophthalmology Center as well as Lymphoma Multidisciplinary Outpatient Clinic. The sites of OAML in our institution were as follows: conjunctiva 59% (73/123), orbit 26.8% (30/123), lacrimal gland 10.6% (13/123), and eyelid 5.3% (7/123), reporting higher incidence of conjunctival lesion than a previous report [1]. OAL was reported to show bilateral involvement in 7 to 24% [19], and our study patients had bilateral lymphoma in 10%.

National Cancer Center Network (NCCN) guideline (v. 2018.4) suggests that chest, abdomen, and pelvic CT and/or FDG PET/CT be done for essential workup [20]. FDG PET/CT is routinely performed for staging of patients with lymphoma at our institution. FDG PET/CT showed positive FDG uptake in 83.3% of non-conjunctival OAML and a detection rate similar to that of orbital MRI. Previous studies with OAML including conjunctival origin reported detection rates between 38.1 and 86% [15, 18, 21]. In our study, FDG uptake intensity varied according to the involved sites: lymphoma of orbit showed significantly higher uptake than lymphoma of eyelid or lacrimal gland. The 16.7% of the lesions without FDG uptake were either located in the eyelids with very small tumor volumes or in the orbit, and distinction from the intense FDG uptake in the ocular muscles was difficult. It remains to be seen how the recent advances with digital PET may improve this sensitivity.

Previous reports showed extraocular involvement in 10–15% of patients with OAL [1]. For primary OAML, Thuro et al. reported extraocular involvement on FDG PET/CT of up to 28.6% of patients [21]. In our study, seven (14%) of primary OAML patients had extraocular involvement detected by FDG PET/CT and were upstaged to stage IIE (*n* = 4) or stage IVE (*n* = 3). There were 8 patients with stage IVE, including the three patients with distant lesions detected by FDG PET/CT. PET/CT did not detect distant lesions in 5 patients: 4 with positive bone marrow involvement and 1 with histologically confirmed multiple skin lesions. Our institution routinely performs bone marrow biopsy at the staging workup, as one report demonstrated positive bone marrow involvement in 12% of OAML patients [21]. Data is lacking to suggest bone marrow biopsy can be omitted in OAML patients with FDG PET/CT imaging performed at diagnosis.

Though FDG PET/CT has limited evidence to replace bone marrow biopsy [22], PET/CT has high sensitivity for detecting nodal and systemic organ involvement. Our findings showing 14% of patients with extraocular involvement further emphasizes the diagnostic value of staging FDG PET/CT. Frontline therapy of OAML is radiation therapy [4, 5], but once there is systemic involvement, chemotherapy is performed. In our study, the treatment plan changed from radiotherapy to chemotherapy in 6 patients. This study included six patients who received both chemotherapy followed by consolidative radiation therapy due to bulky tumor volume or insufficient treatment response of chemotherapy. In one recent study, chemotherapy including rituximab showed good treatment response for both ocular and extraocular lesions [5]. If radiation therapy is performed after orbit MRI without systemic evaluation, extraocular lesions will go undetected and thus untreated. Therefore, FDG PET/CT has a role in initial staging and planning of treatment strategy in patients with non-conjunctival OAML, which has higher rate of systemic involvement and recurrence rate compared with conjunctival OAML [23, 24].

Prognostic value of baseline FDG PET/CT has been reported in previous studies, and we plan on performing analysis of long-term clinical outcome with a larger study population [10, 25, 26].

## Conclusion

In patients with non-conjunctival origin OAML, 14% of patients were upstaged by FDG PET/CT and received systemic chemotherapy instead of radiation therapy. FDG PET/CT should be performed for staging of non-conjunctival origin OAML to guide the therapeutic plan.

## Data Availability

The datasets used in this study are available from the corresponding author on reasonable request.

## Abbreviations

FDG: Fluorodeoxyglucose
MALT: Mucosa-associated lymphoid tissue
MRI: Magnetic resonance imaging
MTV: Metabolic tumor volume
NCCN: National Cancer Center Network
NHL: Non-Hodgkin lymphoma
OAL: Ocular adnexal lymphoma
OAML: Ocular adnexal MALT lymphoma
PET/CT: Positron-emission tomography/computed tomography
SD: Standard deviation
SI: Signal intensity
SUVmax: Maximum standardized uptake value
SUVmean: Mean standardized uptake value
TLG: Total lesion glycolysis
